# Neutralizing and enhancing antibodies against SARS-CoV-2

**DOI:** 10.1186/s41232-022-00233-7

**Published:** 2022-12-05

**Authors:** Yafei Liu, Hisashi Arase

**Affiliations:** 1 Department of Immunochemistry, Research Institute for Microbial Diseases, Osaka University, Osaka, 565-0871 Japan; 2 Laboratory of Immunochemistry, WPI Immunology Frontier Research Center, Osaka University, Osaka, 565-0871 Japan

**Keywords:** SARS-CoV-2, Neutralizing antibody, Enhancing antibody

## Abstract

The high transmissibility and rapid global spread of SARS-CoV-2 since 2019 has led to a huge burden on healthcare worldwide. Anti-SARS-CoV-2 neutralizing antibodies play an important role in not only protecting against infection but also in clearing the virus and are essential to providing long-term immunity. On the other hand, antibodies against the virus are not always protective. With the emergence of SARS-CoV-2 immune escape variants, vaccine design strategies as well as antibody-mediated therapeutic approaches have become more important. We review some of the findings on SARS-CoV-2 antibodies, focusing on both basic research and clinical applications.

## Introduction

In late 2019, the novel severe acute respiratory syndrome coronavirus 2 (SARS-CoV-2) spread from an animal reservoir to humans, causing a respiratory infection known as coronavirus disease 2019 (COVID-19) [1]. The high transmissibility and rapid global spread of SARS-CoV-2 has led to a worldwide COVID-19 pandemic that has continued for more than 2 years, leading to a huge burden on healthcare and society [2]. Although several vaccines and therapeutic antibodies have been approved to prevent COVID-19, effective countermeasures are still needed to control the global COVID-19 pandemic as new immune escape variants continue to emerge.

SARS-CoV-2 is an enveloped, single-stranded, and positive-sense RNA coronavirus [3]. The coronavirus virion is made up of the nucleocapsid (N), membrane (M), envelope (E), and spike (S) structural proteins. The entry steps of the viral particles—encompassing attachment to the host cell membrane and fusion—are mediated by the spike glycoprotein [4]. Spike protein plays the most important role in virus infection. Thus, it is an important target for vaccine and therapeutic antibody development. The SARS-CoV-2 spike protein is cleaved into S1 and S2 subunits, which are responsible for host cell receptor binding and membrane fusion, respectively, mediated through the receptor binding domain (RBD) in the S1 region [5, 6]. The N-terminal domain (NTD) is located on the S1 subunit. Although the functional role of the NTD has not been fully elucidated, it may involve interactions with C-type lectins [7, 8]. Similar to SARS-CoV-1 and some SARS-like coronaviruses, angiotensin-converting enzyme 2 (ACE2) binds to the RBD as a functional cellular receptor for SARS-CoV-2 [5, 9].

The large number of mutations carried in variants poses a major challenge to antibody-mediated immunity. This was initially evident in the SARS-CoV-2 Alpha [10] and Delta variants [11], which both had outbreaks on a global scale, and it became particularly evident in the recently emerged Omicron variant [12], which carries a large number of spike mutations and can evade a wide range of neutralizing antibodies [13]. It is necessary to understand the relationship between this viral evolution and the development and function of anti-SARS-CoV-2 antibodies to inform the development of future vaccines and the effective use of therapeutic antibodies. Here, we review progress in the understanding of antibodies of SARS-CoV-2.

### Anti-RBD-neutralizing antibodies

**Fig. 1:**
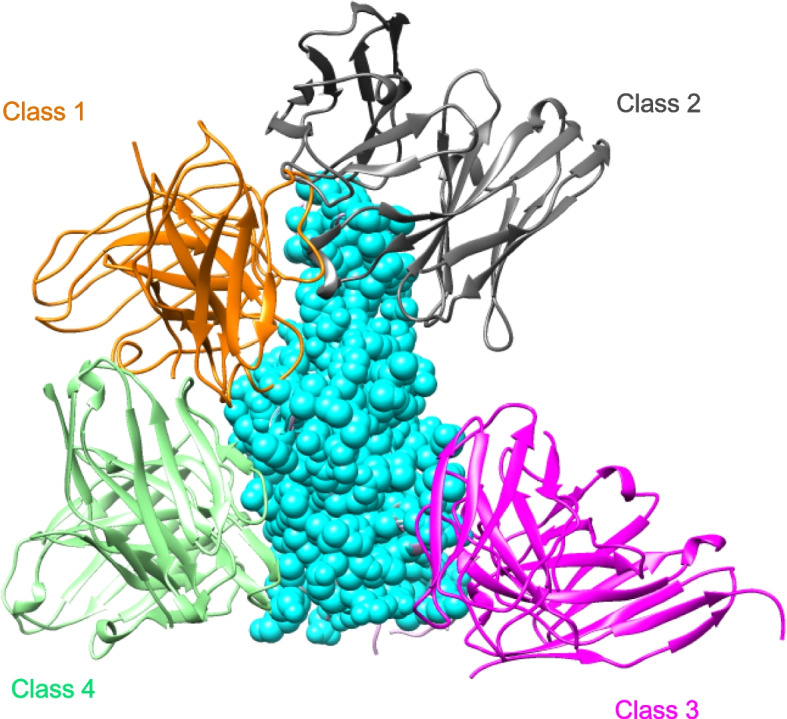
Diagram represents the binding poses of antibodies on the RBD epitope. Class 1 antibodies (C105, PDB: 6XCM) are shown in orange, class 2 (LY-COV555, PDB:7KMG) in gray, class 3 (S309, PDB:7SOC) in magenta, and class 4 (C118, PDB:7RKS) in light sea green

The SARS-CoV-2 spike protein RBD is a critical target for the development of effective COVID-19 antibodies. SARS-CoV-2 RBD-targeting neutralizing antibodies have been extensively studied, and most of the currently developed human neutralizing antibodies are specific to this region. Analysis of the binding between the RBD and RBD-specific neutralizing antibodies, as well as the inhibition of RBD-ACE2 binding by neutralizing antibodies, reveals different antigenic regions on the RBD. Different classification systems for RBDs have been proposed based on data from structural analysis, functional characterization, and/or antigen mapping, and these systems generally classify RBD-neutralizing antibodies into four categories (Fig. 1) [14–19]. RBD-reactive antibodies with the highest in vitro neutralizing potency compete for binding to ACE2 via a targeting receptor binding motif (RBM) [14, 15, 17, 20–24]. In addition to the competitive binding with ACE2, the targeting receptor binding pattern may mimic receptor interactions and trigger premature changes in spike protein conformation to its post-fusion state [25]. Class 1 neutralizing antibodies can only access their epitopes in the open RBD conformation, whereas class 2 RBM-targeting antibodies can bind both the RBD-up and RBD-down positions of the open and closed RBD conformations. By interacting with the adjacent RBD, members of class 2 antibodies can lock the trimer into an “all-RBD-down” closed conformation, thereby preventing RBD-up-dependent ACE2 binding [19, 23, 25–27]. Class 3 RBD-binding antibodies do not interact directly with the RBM, and their epitopes have no or little overlap with the RBM. Although some antibodies, such as sotrovimab (S309) antibodies, belonging to this class do not compete directly with ACE2 binding, they can interfere with receptor binding through steric hindrance [15, 19, 25, 28]. Finally, class 4 RBD-binding antibodies are non-ACE2-competitive RBD antibodies that target conserved epitopes distal to the receptor binding pattern. This class of antibodies has been shown to act by disrupting spike proteins [29–31] and to show broad cross-reactivity with other coronaviruses [32, 33].

Despite its essential function in host cell receptor interactions and membrane fusion, the SARS-CoV-2 spike protein has a remarkable degree of sequence variability. While many of the potential changes in the spike protein can adversely affect viral functions, mutations that increase infectivity and/or transmissibility confer a growth advantage, resulting in antibody resistance that can be advantageous to the evasion of antibody-mediated immune pressure. Notably, several mutations in viral variants of concerns (VoCs) have been identified as key mediators of antibody resistance through in vitro experiments and structure analysis. For example, the E484 mutation can significantly reduce the binding of class 2 neutralizing antibodies, thereby affecting the neutralizing efficacy of the serum against the SARS-CoV-2 variants [19, 34–36]. Similarly, mutations in K417 reduce the binding of class 1 neutralizing antibodies and were selected in the presence of vaccine-induced monoclonal antibodies [35, 37]. VoCs carrying mutations in residues K417 and E484, such as Beta, Gamma, and Omicron, emerged due to the fact that the mutations in residues K417 and E484 reduced sensitivity to antibody-mediated neutralization [12, 38].

Many of the antibodies in clinical development were isolated by antibody cloning methods that rely on the recognition of B cells from COVID-19-convalescent individuals [21, 28, 39–47]. Other antibodies were obtained from immunized mice carrying the human immunoglobulin gene [48, 49]. In addition, readily available samples obtained from humans previously infected with the related SARS-CoV-1 allowed us to identify potent cross-neutralizing antibodies [15, 50, 51]. Experiments in animal models conclusively demonstrated their potential in vivo, as neutralizing antibodies given before or after exposure could prevent or suppress infection [39, 42, 44, 45, 48, 52]. However, the number of mutations has increased as the virus evolves to escape from antibody-mediated immunity. Omicron variant BA.1 contains an unprecedented number of mutations concentrated in the spike gene, with 30 substitutions plus 6 residue deletions and 3 residue insertions, and the extensive mutational burden of Omicron S disrupts the activity of even the most potent neutralizing monoclonal antibodies, leading to severe knockdown or complete loss of the neutralizing capacity of serum from natural infection or vaccination, contributing to increased transmissibility and explosive spread [12, 53].

Before the explosive spread of the Omicron variant around the world in late 2021, we predicted mutations that the Delta variant may acquire in future [54]. According to the RBD mutations that the Delta variant had acquired, we introduced 4 additional mutations (K417N, N439K, E484K, and N501Y) in the RBD of the Delta variant. The neutralizing activity of BNT162b2 immune sera against the hypothetical variant was very much decreased, similar to that observed for the Omicron variant. Although the same Delta variant did not appear, it is noteworthy that Omicron variant BA.5 (K417N, N440K, L452R, T478K, E484A, and N501Y) has acquired quite similar RBD mutations to those in our hypothetical Delta variant (K417N, N439K, L452R, T478K, E484K, and N501Y). A new trial to predict mutations that the SARS-CoV-2 may acquire in future would be an important step in the development of a new vaccine that prevents the appearance of further harmful variants.

Many therapeutic antibodies, such as casirivimab/imdevimab which targeted residue Y453 and G446, lost their effectiveness against BA.5. Thus, more conserved epitopes might be preferential targets as a limited degree of variation suggests restricted mutational capacity. For example, the epitope of the antibody sotrovimab (S309) encompasses highly conserved residues, and structural analysis identified only a small number of single residues conferring potential escape [15, 55, 56]. In addition, structural analysis and epitope and mutational mapping as well as in vitro experiments have provided information on the use of preferential antibody combinations. A combination of antibodies with different epitopes reduces the likelihood of mutations developing that would confer resistance to the antibody. Although it is not clear why the combination of drugs reduces the frequency of mutations, the neutralizing efficacy of the combination is better than that of individual antibodies alone [36, 57–60].

### Anti-NTD-neutralizing antibodies

**Fig. 2:**
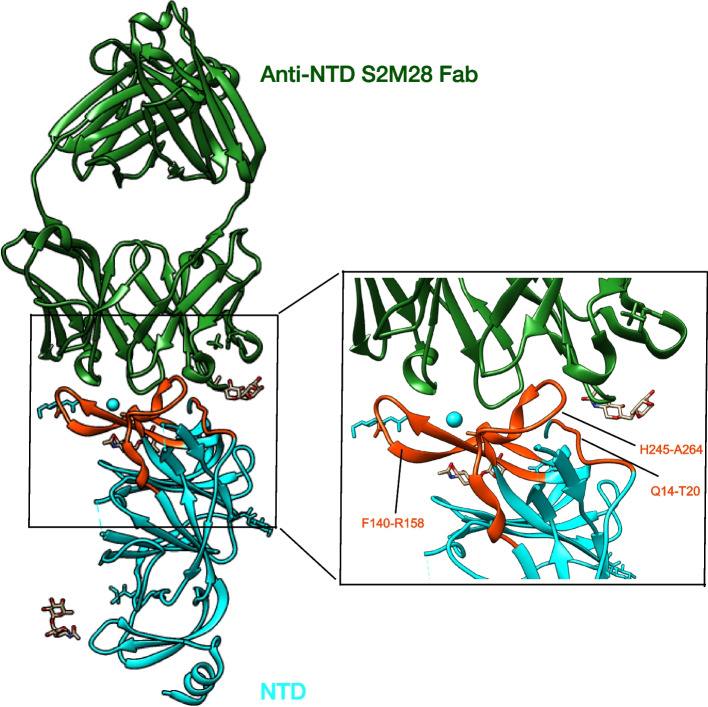
Left figure, ribbon diagrams showing the anti-NTD neutralizing antibody S2M28-binding pose relative to the NTD (PDB: 7LY3). Right figure, zoomed-in views showing the interactions of S2M28 with NTD

SARS-CoV-2 neutralizing activity is not exclusively directed at the RBD. The NTD is another one major site on the S trimer targeted by neutralizing antibodies [23, 61–65]. The NTD has a relatively high glycan density compared with other regions on the spike protein. Based on structural analyzes, binding sites were identified by further structural analysis of a large panel of neutralizing and non-neutralizing monoclonal antibodies [61, 65, 66]. NTD-neutralizing antibodies contact with the N-terminal region at residues 14–20, residues 140–158 (the supersite b-hairpin), and residues 245–264 (the supersite loop). These three regions collectively form an antigenic supersite (Fig. 2). The NTD-neutralizing monoclonal antibodies target the same antigenic supersite, providing examples of convergent solutions to NTD-targeted monoclonal antibody neutralization [67]. Although the precise mechanism underlying NTD-targeted neutralizing activity has not yet been elucidated, several groups have reported that a small subset of monoclonal antibodies that recognize the NTD could neutralize wild-type SARS-CoV-2 by interfering with the fusion of virus and host cell membranes via steric hindrance [51, 61, 64, 67] .

The effectiveness of NTD-neutralizing antibodies has been demonstrated in wild-type and early VoCs such as infected mouse/hamster models [64, 67]. Suryadevara et al. found that the Fab fragments of NTD-neutralizing antibodies lost their neutralizing activity, and *F*(ab’)_2_ forms of NTD-neutralizing antibodies showed decreased neutralizing activity, suggesting that NTD-targeting monoclonal antibodies were capable of mediating Fc effector functions. Further investigation proved that the Fc-mediated activity of NTD-specific monoclonal antibodies did contribute to protection in vivo. In addition, combinations of NTD and RBD monoclonal antibodies demonstrated complementary effects on viral neutralization and Fc effector functions in vitro and yielded potent in vivo prophylactic and therapeutic efficacy [64]. However, studies on the structure of some variants spike NTD have shown that different variant strains can use different strategies to remodel their NTD and evade host immunity. The NTD function does not require specific structural elements, and even the beta chain in the core structure can be rearranged in different ways without reducing virus infectivity. Indeed, research on Omicron variant BA.1 and BA.2 showed that the neutralizing activity of anti-NTD monoclonal antibodies was markedly impaired due to the mutation of G142D and del 143-145 in the spike NTD [68, 69]. Nutalai et al. generated a panel of human monoclonal antibodies from 5 donors who had recovered from BA.1 infection having previously received 2 doses of vaccine. Compared with early pandemic mAb, the proportions of both the RBD- and NTD-binding antibodies were higher among the Omicron monoclonal antibodies. However, among the most 28 potent neutralizing antibodies, only 1 antibody bound to the NTD, suggesting that RBD- but not NTD-neutralizing antibodies play a dominate role against Omicron infection [70]. Although highly related to BA.2, the recently reported Omicron sub-lineages BA.5, which spread globally to replace BA.2. BA.5, contain the 69–70 deletions in the NTD which was also found in BA.1 and Alpha. However, by using the anti-BA.1 monoclonal antibodies for BA.5, the NTD antibody was found to specifically neutralize BA.1, BA.1.1, and BA.3 but not BA.2 or BA.4/5 [71], suggesting that therapeutic antibodies or vaccines should not target the NTD as escaping from anti-NTD antibodies appears to have little cost to the virus.

### Anti-NTD infectivity-enhancing antibody in SARS-CoV-2

Antibody-dependent enhancement (ADE) was first clearly reported in dengue virus infection by Halstead et al. [72]. In cases of ADE, rather than contributing to antiviral immunity, pre-existing antibodies induced viral entry and subsequent infection of host cells, leading to both increased infectivity and virulence. Numerous studies have since identified Fcγ receptors (FcγRs) as the key mediators of ADE in dengue pathogenesis, as they allow for the internalization of multimeric virus-bound IgG and subsequent productive infection. Fc receptor-mediated ADE is restricted to the infection of Fc receptor-expressing cells such as monocytes or macrophages [73]. Whether the ADE of SARS-CoV-2 infection is involved in an immunopathological role that affects clinical outcomes remains controversial. Several studies reported that convalescent serum from SARS-CoV-2 patients contained ADE infection antibody and induced ADE of infection via FcγRIIA and Fcg RIIIA [74, 75]. In another in vitro study, SARS-CoV-2 infection induced antibodies that can cause C1q-mediated ADE [76]. Moreover, Zhou et al. investigated a potent RBD-specific neutralizing antibody. These RBD-neutralizing-IgA antibodies maintained neutralizing activities against SARS-CoV-2 in vitro similar to those of IgG1 antibodies, but IgA antibody increased the amount of infectious virus in the nasal turbinate of Syrian hamsters. In consideration of the potential for antibodies to exacerbate disease through an ADE mechanism, several clinical monoclonal antibodies (etesevimab, AZD8895, and AZD1061) have been designed without FcγR-binding activity. On the other hand, there is no clear evidence for ADE in in vivo studies [64, 77–80], and therapeutic administration of high-dose neutralizing anti-SARS-CoV-2 monoclonal antibody in COVID-19 patients is not associated with disease progression [81–85]. Rachel et al. found that FcγR confers monoclonal antibody-mediated protection rather than induction of ADE. Moreover, the reduced C1q-binding activity showed better therapeutic efficacy compared to wild-type IgG1. The therapeutic efficacy of monoclonal antibodies may be improved by using Fc-engineered antibodies that specifically activate the FcγR pathway [86].

**Fig. 3:**
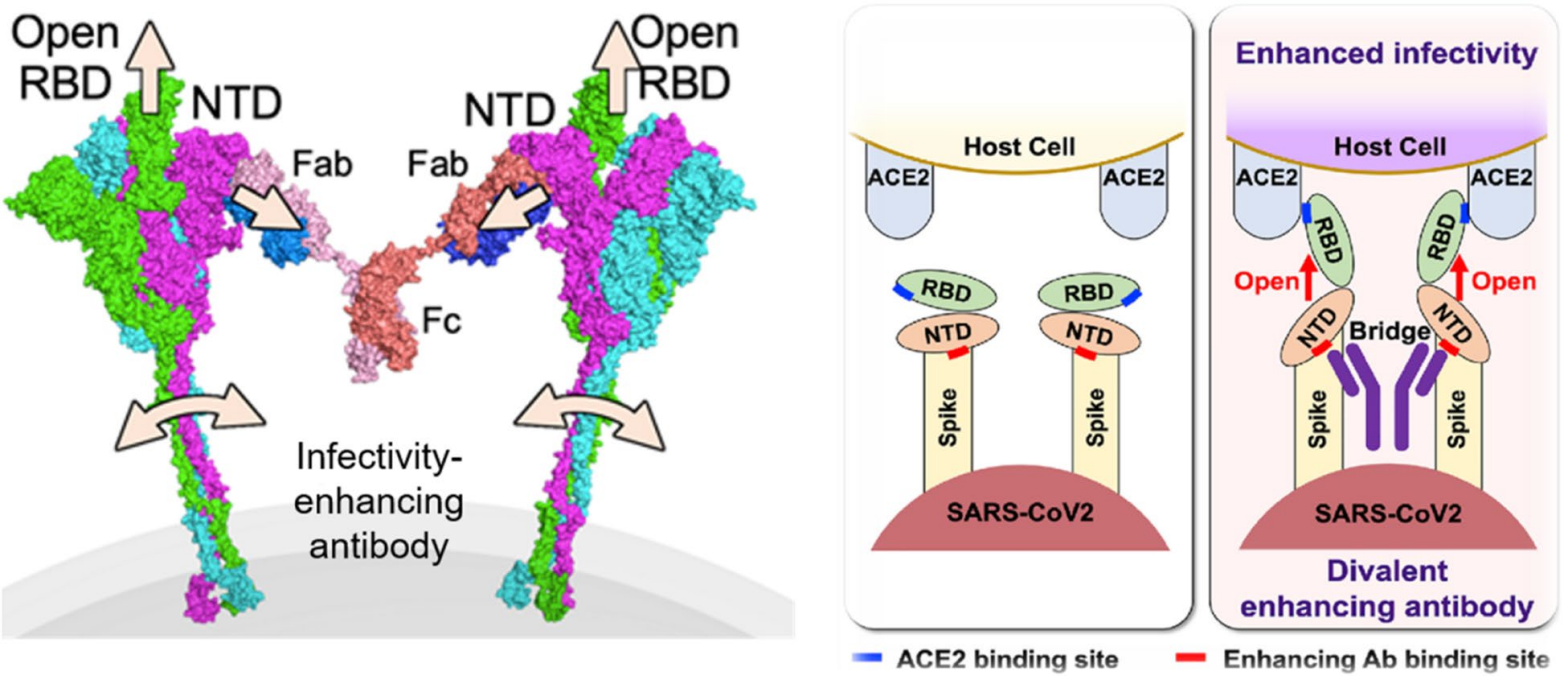
Mechanism underlying the action of infectivity-enhancing antibodies. The antibody-cross-linked NTDs induce open RBDs by being pulled apart, resulting in enhanced infectivity

However, factors other than the Fc-receptor mediated ADE may enhance the infectivity of SARS-CoV-2. Recently, we have found that a different mechanism for ADE has been proposed for SARS-CoV-2, involving antibodies against particular epitopes in the NTD of the S1 component of the spike protein. These infectivity-enhancing antibodies have been shown to induce the RBD to adopt an open conformation, increasing its affinity for ACE2 and thereby enhancing infectivity. Each individual infectivity-enhancing antibody is thought to simultaneously bind to a specific site on the NTD on two adjacent trimeric spike proteins to pull the spike protein into motion (Fig. 3). This result suggests that the RBD adopts an open conformation and becomes highly infectious. Interestingly, these enhancing antibodies have been found in both uninfected and infected individuals, with the infectivity-enhancing antibodies tending to be at high levels in critically ill patients. We also found that the neutralization activity of anti-RBD antibodies is reduced in the presence of infectivity-enhancing antibodies [87]. Moreover, Kimura et al. showed that infectivity-enhancing antibodies enhanced the infectivity of lambada variant more significantly than they did the wild-type [88]. In addition, we examined the Delta pseudovirus with four additional RBD mutations and found that the immune serum enhanced infectivity at low concentrations, probably as the Delta NTD was more sensitive to the infectivity-enhancing antibodies and the RBD- or NTD-neutralizing antibody showed a loss of neutralizing activity [54]. As the number of mutations in VoCs increases, the number of effective neutralizing antibodies will decrease and the balance between enhancing and neutralizing antibodies may be disrupted. However, this conclusion lacks confirmation by in vivo experimental data. Another study, in which anti-SARS-CoV-2 monoclonal antibodies were isolated from a SARS-CoV-2-infected individual, also identified a set of FcγR-independent enhancing antibodies that were similar to our findings in terms of function and specific epitopes in the NTD. Furthermore, the authors used human IgG1 infectivity-enhancing antibodies in vivo in mice and macaques and found no evidence of the enhancement of disease progression. Rather, these antibodies were found to be protective across dose levels. They employed only the wild-type SARS-CoV2 virus to analyze the function of the enhancing antibodies in vitro and in vivo [79]. However, it has been reported that NTD mutations in the Delta variant contribute to increased infectivity [89]. Indeed, enhancing antibodies promote infection of the Delta and Lambda variants more than the wild type SARS-CoV-2 [54, 88]. On the other hand, there are four types of Fc receptors in human and the affinities and functions of these human Fc receptors differ from those of mouse and macaque. Furthermore, because human IgG1 antibodies show the strongest effector function among all subclasses of antibodies, it is possible that the infectivity-enhancing function of the antibodies is canceled by the effector function of IgG1 antibodies. Given that human IgG2 and IgG4 antibodies exhibit lower Fc-receptor-mediated and complement-mediated effector function than IgG1 antibodies, further studies using other subclasses of enhancing antibodies are required to elucidate the exact in vivo function of infectivity-enhancing antibodies.

### Non-NTD-/RBD-binding antibodies

Several neutralizing antibodies binding to the conserved S2 domain have been identified and can reduce viral infection in vivo [90–92]. Several of these SARS-CoV-2 S2-targeting antibodies also cross-react with SARS-CoV-1 S protein, but none of them show neutralizing activity against SARS-CoV-1 infection. In general, the binding affinity and neutralizing activity of non-RBD and non-NTD Abs against SARS-CoV-2 infection are lower than those of neutralizing antibodies targeting the S protein NTD or RBD of SARS-CoV-2. There have been few reports of their detection on VoCs with S2 antibody. As mentioned above, among the neutralizing antibodies isolated from Omicron-infected patients, there were no potent neutralizing antibodies binding to spike S2. Although modest neutralizing potency may limit their potential for clinical application, the high degree of cross-reactivity makes S2-targeted antibodies informative in pan-betacoronavirus vaccine design [90, 92, 93].

## Conclusion

The high incidence of SARS-CoV-2 infection and the pressure of immune-mediated selection will drive the continuous evolution of the virus and the emergence of SARS-CoV-2 immune escape variants. This will severely impair antibody-mediated immunity and impact the effect of current therapeutic antibodies. Thus, predicting virus mutations can facilitate vaccine development. The development of antibodies targeting conserved epitopes and the use of combinations of neutralizing antibodies with different epitopes could improve the effectiveness of neutralizing antibodies. Several NTD antibodies effectively neutralize viral infections, and the NTD-antibody binding site is completely different from that of the RBD. However, specific structural elements are not required to maintain NTD function, and even the core structural sequence of the NTD is rearranged under antibody-mediated selection pressure. On the other hand, despite the lack of in vivo data, anti-NTD-enhancing antibodies increase the risk of therapeutic antibodies or universal vaccines against NTD. With this in mind, a chimeric RBD-dimer vaccine was recently developed to adapt to the currently prevalent variants although they possess lower T cell epitopes [94]. In the future, more in-depth research and understanding of anti-NTD and anti-RBD antibodies is needed to address more diverse and evolving viral threats.

## Data Availability

Not applicable.

## Abbreviations

SARS-CoV-2: Severe acute respiratory syndrome coronavirus 2
COVID-19: Coronavirus disease 2019
RBD: Receptor-binding domain
NTD: The N-terminal domain
ACE2: Angiotensin-converting enzyme 2
RBM: Receptor-binding motif
VoCs: Viral variants of concerns
ADE: Antibody-dependent enhancement
FcγRs: Fcγ receptors

## References

[1] Zhu N, Zhang D, Wang W, Li X, Yang B, Song J (2020). A novel coronavirus from patients with pneumonia in China, 2019. N Engl J Med.

[2] Chotpitayasunondh T, Fischer TK, Heraud JM, Hurt AC, Monto AS, Osterhaus A (2021). Influenza and COVID-19: what does co-existence mean?. Influenza Other Respi Viruses.

[3] Wang N, Shang J, Jiang S, Du L (2020). Subunit vaccines against emerging pathogenic human coronaviruses. Front Microbiol.

[4] Hu B, Guo H, Zhou P, Shi ZL (2021). Characteristics of SARS-CoV-2 and COVID-19. Nat Rev Microbiol.

[5] Shang J, Ye G, Shi K, Wan YS, Luo CM, Aihara H (2020). Structural basis of receptor recognition by SARS-CoV-2. Nature.

[6] Xia S, Liu MQ, Wang C, Xu W, Lan QS, Feng SL (2020). Inhibition of SARS-CoV-2 (previously 2019-nCoV) infection by a highly potent pan-coronavirus fusion inhibitor targeting its spike protein that harbors a high capacity to mediate membrane fusion. Cell Res.

[7] Seyran M, Takayama K, Uversky VN, Lundstrom K, Palu G, Sherchan SP (2021). The structural basis of accelerated host cell entry by SARS-CoV-2 dagger. FEBS J.

[8] Soh WT, Liu Y, Nakayama EE, Ono C, Torii S, Nakagami H, et al. The N-terminal domain of spike glycoprotein mediates SARS-CoV-2 infection by associating with L-SIGN and DCSIGN. Preprint at BioRxiv. 2020. 10.1101/2020.11.05.369264.

[9] Zhou P, Yang XL, Wang XG, Hu B, Zhang L, Zhang W (2020). A pneumonia outbreak associated with a new coronavirus of probable bat origin. Nature.

[10] Supasa P, Zhou D, Dejnirattisai W, Liu C, Mentzer AJ, Ginn HM (2021). Reduced neutralization of SARS-CoV-2 B.1.1.7 variant by convalescent and vaccine sera. Cell.

[11] Liu C, Ginn HM, Dejnirattisai W, Supasa P, Wang B, Tuekprakhon A (2021). Reduced neutralization of SARS-CoV-2 B.1.617 by vaccine and convalescent serum. Cell.

[12] Dejnirattisai W, Huo J, Zhou D, Zahradnik J, Supasa P, Liu C (2022). SARS-CoV-2 Omicron-B.1.1.529 leads to widespread escape from neutralizing antibody responses. Cell.

[13] Viana R, Moyo S, Amoako DG, Tegally H, Scheepers C, Althaus CL (2022). Rapid epidemic expansion of the SARS-CoV-2 Omicron variant in southern Africa. Nature.

[14] Dejnirattisai W, Zhou D, Ginn HM, Duyvesteyn HME, Supasa P, Case JB (2021). The antigenic anatomy of SARS-CoV-2 receptor binding domain. Cell.

[15] Pinto D, Park YJ, Beltramello M, Walls AC, Tortorici MA, Bianchi S (2020). Cross-neutralization of SARS-CoV-2 by a human monoclonal SARS-CoV antibody. Nature.

[16] Hastie KM, Li H, Bedinger D, Schendel SL, Dennison SM, Li K (2021). Defining variant-resistant epitopes targeted by SARS-CoV-2 antibodies: a global consortium study. Science.

[17] Piccoli L, Park YJ, Tortorici MA, Czudnochowski N, Walls AC, Beltramello M (2020). Mapping neutralizing and immunodominant sites on the SARS-CoV-2 spike receptor-binding domain by structure-guided high-resolution serology. Cell.

[18] Brouwer PJM, Caniels TG, van der Straten K, Snitselaar JL, Aldon Y, Bangaru S (2020). Potent neutralizing antibodies from COVID-19 patients define multiple targets of vulnerability. Science.

[19] Barnes CO, Jette CA, Abernathy ME, Dam KA, Esswein SR, Gristick HB (2020). SARS-CoV-2 neutralizing antibody structures inform therapeutic strategies. Nature.

[20] Yuan M, Wu NC, Zhu X, Lee CD, So RTY, Lv H (2020). A highly conserved cryptic epitope in the receptor binding domains of SARS-CoV-2 and SARS-CoV. Science.

[21] Ju B, Zhang Q, Ge J, Wang R, Sun J, Ge X (2020). Human neutralizing antibodies elicited by SARS-CoV-2 infection. Nature.

[22] Wu Y, Wang F, Shen C, Peng W, Li D, Zhao C (2020). A noncompeting pair of human neutralizing antibodies block COVID-19 virus binding to its receptor ACE2. Science.

[23] Liu L, Wang P, Nair MS, Yu J, Rapp M, Wang Q (2020). Potent neutralizing antibodies against multiple epitopes on SARS-CoV-2 spike. Nature.

[24] Barnes CO, West AP, Huey-Tubman KE, Hoffmann MAG, Sharaf NG, Hoffman PR (2020). Structures of human antibodies bound to SARS-CoV-2 spike reveal common epitopes and recurrent features of antibodies. Cell.

[25] Lempp FA, Soriaga LB, Montiel-Ruiz M, Benigni F, Noack J, Park YJ (2021). Lectins enhance SARS-CoV-2 infection and influence neutralizing antibodies. Nature.

[26] Scheid JF, Barnes CO, Eraslan B, Hudak A, Keeffe JR, Cosimi LA (2021). B cell genomics behind cross-neutralization of SARS-CoV-2 variants and SARS-CoV. Cell.

[27] Tortorici MA, Beltramello M, Lempp FA, Pinto D, Dang HV, Rosen LE (2020). Ultrapotent human antibodies protect against SARS-CoV-2 challenge via multiple mechanisms. Science.

[28] Hansen J, Baum A, Pascal KE, Russo V, Giordano S, Wloga E (2020). Studies in humanized mice and convalescent humans yield a SARS-CoV-2 antibody cocktail. Science.

[29] Wrobel AG, Benton DJ, Hussain S, Harvey R, Martin SR, Roustan C (2020). Antibody-mediated disruption of the SARS-CoV-2 spike glycoprotein. Nat Commun.

[30] Huo J, Zhao Y, Ren J, Zhou D, Duyvesteyn HME, Ginn HM (2020). Neutralization of SARS-CoV-2 by destruction of the prefusion spike. Cell Host Microbe.

[31] Yuan M, Liu H, Wu NC, Lee CD, Zhu X, Zhao F (2020). Structural basis of a shared antibody response to SARS-CoV-2. Science.

[32] Jette CA, Cohen AA, Gnanapragasam PNP, Muecksch F, Lee YE, Huey-Tubman KE (2021). Broad cross-reactivity across sarbecoviruses exhibited by a subset of COVID-19 donor-derived neutralizing antibodies. Cell Rep.

[33] Martinez DR, Schafer A, Leist SR, Li D, Gully K, Yount B (2021). Prevention and therapy of SARS-CoV-2 and the B.1.351 variant in mice. Cell Rep.

[34] Greaney AJ, Loes AN, Crawford KHD, Starr TN, Malone KD, Chu HY (2021). Comprehensive mapping of mutations in the SARS-CoV-2 receptor-binding domain that affect recognition by polyclonal human plasma antibodies. Cell Host Microbe.

[35] Greaney AJ, Starr TN, Barnes CO, Weisblum Y, Schmidt F, Caskey M (2021). Mapping mutations to the SARS-CoV-2 RBD that escape binding by different classes of antibodies. Nat Commun.

[36] Liu Z, VanBlargan LA, Bloyet LM, Rothlauf PW, Chen RE, Stumpf S (2021). Identification of SARS-CoV-2 spike mutations that attenuate monoclonal and serum antibody neutralization. Cell Host Microbe.

[37] Wang Z, Schmidt F, Weisblum Y, Muecksch F, Barnes CO, Finkin S (2021). mRNA vaccine-elicited antibodies to SARS-CoV-2 and circulating variants. Nature.

[38] Carreno JM, Alshammary H, Tcheou J, Singh G, Raskin AJ, Kawabata H (2022). Activity of convalescent and vaccine serum against SARS-CoV-2 Omicron. Nature.

[39] Zost SJ, Gilchuk P, Case JB, Binshtein E, Chen RE, Nkolola JP (2020). Potently neutralizing and protective human antibodies against SARS-CoV-2. Nature.

[40] Zost SJ, Gilchuk P, Chen RE, Case JB, Reidy JX, Trivette A (2020). Rapid isolation and profiling of a diverse panel of human monoclonal antibodies targeting the SARS-CoV-2 spike protein. Nat Med.

[41] Robbiani DF, Gaebler C, Muecksch F, Lorenzi JCC, Wang Z, Cho A (2020). Convergent antibody responses to SARS-CoV-2 in convalescent individuals. Nature.

[42] Shi R, Shan C, Duan X, Chen Z, Liu P, Song J (2020). A human neutralizing antibody targets the receptor-binding site of SARS-CoV-2. Nature.

[43] Andreano E, Nicastri E, Paciello I, Pileri P, Manganaro N, Piccini G (2021). Extremely potent human monoclonal antibodies from COVID-19 convalescent patients. Cell.

[44] Cao Y, Su B, Guo X, Sun W, Deng Y, Bao L (2020). Potent neutralizing antibodies against SARS-CoV-2 identified by high-throughput single-cell sequencing of convalescent patients’ B cells. Cell.

[45] Du S, Cao Y, Zhu Q, Yu P, Qi F, Wang G (2020). Structurally resolved SARS-CoV-2 antibody shows high efficacy in severely infected hamsters and provides a potent cocktail pairing strategy. Cell.

[46] Gieselmann L, Kreer C, Ercanoglu MS, Lehnen N, Zehner M, Schommers P (2021). Effective high-throughput isolation of fully human antibodies targeting infectious pathogens. Nat Protoc.

[47] Guo Y, Huang L, Zhang G, Yao Y, Zhou H, Shen S (2021). A SARS-CoV-2 neutralizing antibody with extensive spike binding coverage and modified for optimal therapeutic outcomes. Nat Commun.

[48] Bertoglio F, Fuhner V, Ruschig M, Heine PA, Abassi L, Klunemann T (2021). A SARS-CoV-2 neutralizing antibody selected from COVID-19 patients binds to the ACE2-RBD interface and is tolerant to most known RBD mutations. Cell Rep.

[49] Zhu L, Deng YQ, Zhang RR, Cui Z, Sun CY, Fan CF (2021). Double lock of a potent human therapeutic monoclonal antibody against SARS-CoV-2. Natl Sci Rev.

[50] Wec AZ, Wrapp D, Herbert AS, Maurer DP, Haslwanter D, Sakharkar M (2020). Broad neutralization of SARS-related viruses by human monoclonal antibodies. Science.

[51] Rappazzo CG, Tse LV, Kaku CI, Wrapp D, Sakharkar M, Huang D (2021). Broad and potent activity against SARS-like viruses by an engineered human monoclonal antibody. Science.

[52] Baum A, Ajithdoss D, Copin R, Zhou A, Lanza K, Negron N (2020). REGN-COV2 antibodies prevent and treat SARS-CoV-2 infection in rhesus macaques and hamsters. Science.

[53] Cele S, Jackson L, Khoury DS, Khan K, Moyo-Gwete T, Tegally H (2022). Omicron extensively but incompletely escapes Pfizer BNT162b2 neutralization. Nature.

[54] Liu Y, Arase N, Kishikawa J-i, Hirose M, Li S, Tada A, et al. The SARS-CoV-2 Delta variant is poised to acquire complete resistance to wild-type spike vaccines. preprint at BioRxiv. 2022. 10.1101/2021.08.22.457114.

[55] Cathcart AL, Havenar-Daughton C, Lempp FA, Ma D, Schmid MA, Agostini ML, et al. The dual function monoclonal antibodies VIR-7831 and VIR-7832 demonstrate potent in vitro and in vivo activity against SARS-CoV-2. Preprint at BioRxiv. 2022. 10.1101/2021.03.09.434607.

[56] Starr TN, Czudnochowski N, Liu Z, Zatta F, Park YJ, Addetia A (2021). SARS-CoV-2 RBD antibodies that maximize breadth and resistance to escape. Nature.

[57] Weisblum Y, Schmidt F, Zhang F, DaSilva J, Poston D, Lorenzi JC, et al. Escape from neutralizing antibodies by SARS-CoV-2 spike protein variants. Elife. 2020;9:e61312. 10.7554/eLife.61312 PMC7723407 33112236

[58] Baum A, Fulton BO, Wloga E, Copin R, Pascal KE, Russo V (2020). Antibody cocktail to SARS-CoV-2 spike protein prevents rapid mutational escape seen with individual antibodies. Science.

[59] Starr TN, Greaney AJ, Dingens AS, Bloom JD (2021). Complete map of SARS-CoV-2 RBD mutations that escape the monoclonal antibody LY-CoV555 and its cocktail with LY-CoV016. Cell Rep Med.

[60] Starr TN, Greaney AJ, Addetia A, Hannon WW, Choudhary MC, Dingens AS (2021). Prospective mapping of viral mutations that escape antibodies used to treat COVID-19. Science.

[61] Cerutti G, Guo Y, Zhou T, Gorman J, Lee M, Rapp M (2021). Potent SARS-CoV-2 neutralizing antibodies directed against spike N-terminal domain target a single supersite. Cell Host Microbe.

[62] Chi X, Yan R, Zhang J, Zhang G, Zhang Y, Hao M (2020). A neutralizing human antibody binds to the N-terminal domain of the Spike protein of SARS-CoV-2. Science.

[63] Wang Z, Muecksch F, Cho A, Gaebler C, Hoffmann HH, Ramos V (2022). Analysis of memory B cells identifies conserved neutralizing epitopes on the N-terminal domain of variant SARS-Cov-2 spike proteins. Immunity.

[64] Suryadevara N, Shrihari S, Gilchuk P, VanBlargan LA, Binshtein E, Zost SJ (2021). Neutralizing and protective human monoclonal antibodies recognizing the N-terminal domain of the SARS-CoV-2 spike protein. Cell.

[65] Voss WN, Hou YJ, Johnson NV, Delidakis G, Kim JE, Javanmardi K (2021). Prevalent, protective, and convergent IgG recognition of SARS-CoV-2 non-RBD spike epitopes. Science.

[66] Cao Y, Yisimayi A, Bai Y, Huang W, Li X, Zhang Z (2021). Humoral immune response to circulating SARS-CoV-2 variants elicited by inactivated and RBD-subunit vaccines. Cell Res.

[67] McCallum M, De Marco A, Lempp F, Tortorici MA, Pinto D, Walls AC (2021). N-terminal domain antigenic mapping reveals a site of vulnerability for SARS-CoV-2. Cell.

[68] Iketani S, Liu L, Guo Y, Liu L, Chan JF, Huang Y (2022). Antibody evasion properties of SARS-CoV-2 Omicron sublineages. Nature.

[69] Liu L, Iketani S, Guo Y, Chan JF, Wang M, Liu L (2022). Striking antibody evasion manifested by the Omicron variant of SARS-CoV-2. Nature.

[70] Nutalai R, Zhou D, Tuekprakhon A, Ginn HM, Supasa P, Liu C (2022). Potent cross-reactive antibodies following Omicron breakthrough in vaccinees. Cell.

[71] Tuekprakhon A, Nutalai R, Dijokaite-Guraliuc A, Zhou D, Ginn HM, Selvaraj M (2022). Antibody escape of SARS-CoV-2 Omicron BA.4 and BA.5 from vaccine and BA.1 serum. Cell.

[72] Halstead SB, Chow JS, Marchette NJ (1973). Immunological enhancement of dengue virus replication. Nat New Biol.

[73] Bournazos S, Gupta A, Ravetch JV (2020). The role of IgG Fc receptors in antibody-dependent enhancement. Nat Rev Immunol.

[74] Maemura T, Kuroda M, Armbrust T, Yamayoshi S, Halfmann PJ, Kawaoka Y (2021). Antibody-dependent enhancement of SARS-CoV-2 infection is mediated by the IgG receptors FcgammaRIIA and FcgammaRIIIA but does not contribute to aberrant cytokine production by macrophages. mBio.

[75] Wang Z, Deng T, Zhang Y, Niu W, Nie Q, Yang S (2022). ACE2 can act as the secondary receptor in the FcgammaR-dependent ADE of SARS-CoV-2 infection. iScience.

[76] Okuya K, Hattori T, Saito T, Takadate Y, Sasaki M, Furuyama W (2022). Multiple routes of antibody-dependent enhancement of SARS-CoV-2 infection. Microbiol Spectr.

[77] Winkler ES, Gilchuk P, Yu JS, Bailey AL, Chen RE, Chong ZL (2021). Human neutralizing antibodies against SARS-CoV-2 require intact Fc effector functions for optimal therapeutic protection. Cell.

[78] Schafer A, Muecksch F, Lorenzi JCC, Leist SR, Cipolla M, Bournazos S, et al. Antibody potency, effector function, and combinations in protection and therapy for SARS-CoV-2 infection in vivo. J Exp Med. 2021;218(3):e20201993. 10.1084/jem.20201993 PMC7673958 33211088

[79] Ullah I, Prevost J, Ladinsky MS, Stone H, Lu ML, Anand SP (2021). Live imaging of SARS-CoV-2 infection in mice reveals that neutralizing antibodies require Fc function for optimal efficacy. Immunity.

[80] Li D, Edwards RJ, Manne K, Martinez DR, Schafer A, Alam SM (2021). In vitro and in vivo functions of SARS-CoV-2 infection-enhancing and neutralizing antibodies. Cell.

[81] Joyner MJ, Wright RS, Fairweather D, Senefeld JW, Bruno KA, Klassen SA et al: Early safety indicators of COVID-19 convalescent plasma in 5000 patients. J Clin Invest. 2020;130(9):4791-4797. 10.1172/JCI140200 PMC7456238 32525844

[82] Gottlieb RL, Nirula A, Chen P, Boscia J, Heller B, Morris J (2021). Effect of bamlanivimab as monotherapy or in combination with etesevimab on viral load in patients with mild to moderate COVID-19: a randomized clinical trial. Jama.

[83] Weinreich DM, Sivapalasingam S, Norton T, Ali S, Gao H, Bhore R (2021). REGN-COV2, a neutralizing antibody cocktail, in outpatients with COVID-19. N Engl J Med.

[84] Chen P, Nirula A, Heller B, Gottlieb RL, Boscia J, Morris J (2021). SARS-CoV-2 neutralizing antibody LY-CoV555 in outpatients with COVID-19. N Engl J Med.

[85] Lundgren JD, Grund B, Barkauskas CE, Holland TL, Gottlieb RL, Group A-TL-CS (2021). A neutralizing monoclonal antibody for hospitalized patients with COVID-19. N Engl J Med.

[86] Yamin R, Jones AT, Hoffmann HH, Schafer A, Kao KS, Francis RL (2021). Fc-engineered antibody therapeutics with improved anti-SARS-CoV-2 efficacy. Nature.

[87] Liu Y, Soh WT, Kishikawa JI, Hirose M, Nakayama EE, Li S (2021). An infectivity-enhancing site on the SARS-CoV-2 spike protein targeted by antibodies. Cell.

[88] Kimura I, Kosugi Y, Wu J, Zahradnik J, Yamasoba D, Butlertanaka EP (2022). The SARS-CoV-2 Lambda variant exhibits enhanced infectivity and immune resistance. Cell Rep.

[89] Mishra T, Dalavi R, Joshi G, Kumar A, Pandey P, Shukla S, et al. SARS-CoV-2 spike E156G/Delta157-158 mutations contribute to increased infectivity and immune escape. Life Sci Alliance. 2022;5(7):e202201415. 10.26508/lsa.202201415 PMC8927725 35296517

[90] Pinto D, Sauer MM, Czudnochowski N, Low JS, Tortorici MA, Housley MP (2021). Broad betacoronavirus neutralization by a stem helix-specific human antibody. Science.

[91] Jennewein MF, MacCamy AJ, Akins NR, Feng J, Homad LJ, Hurlburt NK (2021). Isolation and characterization of cross-neutralizing coronavirus antibodies from COVID-19+ subjects. Cell Rep.

[92] Zhou P, Yuan M, Song G, Beutler N, Shaabani N, Huang D (2022). A human antibody reveals a conserved site on beta-coronavirus spike proteins and confers protection against SARS-CoV-2 infection. Sci Transl Med.

[93] Ng KW, Faulkner N, Finsterbusch K, Wu M, Harvey R, Hussain S (2022). SARS-CoV-2 S2-targeted vaccination elicits broadly neutralizing antibodies. Sci Transl Med.

[94] Xu K, Gao P, Liu S, Lu S, Lei W, Zheng T (2022). Protective prototype-Beta and Delta-Omicron chimeric RBD-dimer vaccines against SARS-CoV-2. Cell.

